# Automated TruTip nucleic acid extraction and purification from raw sputum

**DOI:** 10.1371/journal.pone.0199869

**Published:** 2018-07-05

**Authors:** Nitu Thakore, Ryan Norville, Molly Franke, Roger Calderon, Leonid Lecca, Michael Villanueva, Megan B. Murray, Christopher G. Cooney, Darrell P. Chandler, Rebecca C. Holmberg

**Affiliations:** 1 Akonni Biosystems, Inc., Frederick, Maryland, United States of America; 2 Department of Global Health and Social Medicine, Harvard Medical School, Boston, Massachusetts, United States of America; 3 Socios En Salud Sucursal Perú, Carabayllo, Lima, Peru; 4 Laboratorios Medicos Especialido, Juarez, Mexico; GGD Amsterdam, NETHERLANDS

## Abstract

Automated nucleic acid extraction from primary (raw) sputum continues to be a significant technical challenge for molecular diagnostics. In this work, we developed a prototype open-architecture, automated nucleic acid workstation that includes a mechanical homogenization and lysis function integrated with heating and TruTip purification; optimized an extraction protocol for raw sputum; and evaluated system performance on primary clinical specimens. Eight samples could be processed within 70 min. The system efficiently homogenized primary sputa and doubled nucleic acid recovery relative to an automated protocol that did not incorporate sample homogenization. Nucleic acid recovery was at least five times higher from raw sputum as compared to that of matched sediments regardless of smear or culture grade, and the automated workstation reproducibly recovered PCR-detectable DNA to at least 80 CFU mL^-1^ raw sputum. *M*. *tuberculosis* DNA was recovered and detected from 122/123 (99.2%) and 124/124 (100%) primary sputum and sediment extracts, respectively. There was no detectable cross-contamination across 53 automated system runs and amplification or fluorescent inhibitors (if present) were not detectable. The open fluidic architecture of the prototype automated workstation yields purified sputum DNA that can be used for any molecular diagnostic test. The ability to transfer TruTip protocols between personalized, on-demand pipetting tools and the fully automated workstation also affords public health agencies an opportunity to standardize sputum nucleic acid sample preparation procedures, reagents, and quality control across multiple levels of the health care system.

## Introduction

Nucleic acid technologies are having a significant impact on the diagnosis, treatment, and control of drug-resistant *Mycobacterium tuberculosis* (*M*. *tuberculosis*), and there is a growing emphasis on developing and deploying molecular diagnostics outside of reference laboratories and closer to the point of need (e.g., [1, 2]). Most tests for diagnosing pulmonary tuberculosis are performed on sputum [2–4], which as clinical sample matrix is also important for managing cyctic fibrosis patients [5, 6]; detecting and diagnosing respiratory viruses [7, 8], pneumonia [9], and other lower respiratory tract infections [10]; and even lung cancer diagnosis or screening [11].

At the intersection of a clinical specimen and the molecular diagnostic is the oft-overlooked problem of nucleic acid sample preparation. While there is no shortage of manual sample preparation methods for use in reference or hospital laboratories [12–19], the specific combination of *M*. *tuberculosis* and primary (raw) sputum presents a number of technical and logistical challenges for automated sample preparation systems and sample-to-answer diagnostic devices because sputum is a complex, viscous, clumpy and non-homogenous sample that contains mucus, human cells, non-target bacteria and viruses, blood and pus [17, 20, 21], and the *M*. *tuberculosis* cell wall is difficult to lyse with chemical-based nucleic acid extraction kits [22–25]. For these reasons, mechanical sample homogenization and cell lysis (sonication, bead beating or bead blending) are often used to prepare sputum samples for nucleic acid tests, as these methods tend to improve DNA recovery relative to purely chemical or enzymatic processes [12, 17, 19, 22, 23, 26].

Unfortunately, commercially available sample preparation devices lack an integrated mechanical homogenization and lysis function that is important for extracting *M*. *tuberculosis* DNA from raw sputum, irrespective of the sample preparation chemistry or extraction method (beads, columns, filters). Even in high resource settings and within the context of centralized testing labs, then, there is still a need for a simple, flexible, automated nucleic acid extraction system that can process raw sputum. The objectives of this work were therefore to 1) design and develop a prototype benchtop, automated nucleic acid workstation with an integrated mechanical homogenizer/lysis function that would meet many of the user needs or requirements defined by the TB community (as summarized in [2, 4]); 2) optimize an automated extraction protocol for raw sputum that generates purified DNA suitable for down-stream nucleic acid amplification and analysis; 3) establish analytical performance metrics for the system and method; and 4) evaluate the system behavior and potential clinical utility on primary sputum specimens, with an emphasis on known or suspected TB-positive patients.

## Materials and methods

### Reference materials and cell culture

Purified *M*. *tuberculosis* H37Ra genomic DNA was purchased from the American Type Culture Collection (ATCC, Manassas, VA; #25177D-5), re-suspended in molecular biology grade water, and quantified on a NanoDrop 3300 fluorometer and frozen at -20°C until use. *M*. *tuberculosis* H37Ra cells were purchased from ATCC (#25177) and grown on solid culture LJ slants (Becton Dickenson, Sparks, MD; catalogue #220908) for up to 8 weeks. Individual colonies were further propagated in 7H9 broth (BD, catalogue #221832) containing glycerol and 0.05% Tween-80 to a turbidity of approximately 1 McFarland (~2 x 10^8^ cells mL^-1^). Cultured cells were de-clumped by vortexing for 1 min in the presence of 3 mm glass beads [27], serially diluted in 7H9 broth, and quantified in CFU mL^-1^ by plating cell dilutions on 7H10 agar (BD, #221174). Quantified cell suspensions and dilutions were frozen at -20°C until use.

De-identified, TB-negative sputum remnants from (symptomatic) cystic fibrosis patients were purchased from BioreclamationIVT (Baltimore, MD) and stored at -20°C. Sputum remnants (3 mL each) were heterogenous in color, viscosity, and clumpiness. Unprocessed remnants were used to prepare spiked samples for system development, assay optimization, and analytical performance tests.

### Automated TruTip materials and reagents

The TruTip is based on a rigid, monolithic, highly porous silica binding matrix embedded within an aerosol-resistant pipette tip, as described elsewhere [28]. TruTip procedures and reagents are predicated on the well-established Boom chemistry [29] and involve a chaotropic lysis/binding buffer, wash buffer(s), and a low-salt elution buffer. All TruTip and automated workstation consumables, reagents, and materials were manufactured by Akonni Biosystems. 1.2 mL SPT TruTips (# 302–80021) were used for all experiments, and starting reagents were taken from the Akonni TruTip gDNA Blood Extraction Kit (# 300–20341, and as described in [30]). Stand-alone 1 mL flat-bottomed polyethylene sample lysis tubes were prepared with 0.3 g inert particles and magnetic stir disc (# 402–00100), and stand-alone 2.2 mL heater tubes (# 402–002) were pre-filled with the guanidium-based lysis buffer. Thereafter, each row of a polypropylene 96-well deep-well reagent plate (USA Scientific #1896–2110) was pre-filled with all remaining reagents necessary to perform nucleic acid binding, wash, and elution functions. Reagent plates were sealed with a pierceable foil seal for routine sample processing.

### Clinical samples

Patients receiving care at three health centers in Lima, Peru and whose sputa tested positive for acid fast bacilli (AFB) by Ziehl Neelsen smear microscopy were invited to provide an additional sample for research purposes. The protocol was approved by the Harvard Faculty of Medicine institutional review board and by the ethics committee of the Peru National Institutes of Health. All participants provided written informed consent before additional samples were collected. All primary specimens samples (~5–10 mL each) were collected and handled under BSL-2 controls, while all *M*. *tuberculosis* cultures were performed under BSL-3 controls at Socios En Salud Sucursal Perú. Approximately 2 mL of each primary sputum was decontaminated in freshly prepared 2% NaOH—0.25% n-acetyl-L-cysteine (NALC) for 15 min at room temperature, and neutralized by diluting to 50 mL total volume in phosphate buffered saline (PBS). Cells were collected by centrifugation at 3,000 x g for 30 min and re-suspended in 1.5 mL PBS, after which 0.2 mL of the decontaminated sediment suspension was inoculated onto replicate solid culture LJ slants. Slants were incubated at 37°C for up to 8 weeks, and smear microscopy was performed on culture positive samples to confirm the presence of *M*. *tuberculosis*.

A subset of samples was processed on the automated TruTip workstation on-site in Lima, Peru. The remaining volume of de-identified primary sputa and their paired sediment samples were shipped to Akonni on dry ice under an approved CDC import permit and stored at -80°C until use.

### Automated nucleic acid extraction

Numerous, iterative instrument designs and experiments were performed to arrive at the prototype workstation and optimized protocol described here. The prototype workstation and design/engineering rationale are described in the results and discussion, below.

Before extraction, approximately 1 mL of raw sputum was liquefied with 80 μL of an enzymatic liquefaction buffer for 20 min at 56°C. Thereafter, 0.5 mL liquefied sputum or sediment was manually transferred to a sample lysis tube (within the confines of a biosafety cabinet) and sealed with a pierceable foil cover before loading lysis tubes onto the automated workstation. The optimized, automated protocol is outlined in Table 1. Eight samples could be extracted in parallel within 70 min of launching the automated program. Paired sediment suspensions (500 μL) were processed in identical fashion except that sediments did not undergo the enzymatic liquefaction step. Water blanks were used as independent, external negative controls for each run. Purified nucleic acids were eluted from the TruTip in 100 μL of 10 mM Tris-HCl (pH 8.0). Purified DNA was stored at -20°C until use.

**Table 1:** Optimized, automated TruTip extraction protocol for liquefied, raw sputum.

Operation	Programmed parameter(s)
Sample homogenization	10 min magnetic vortexing at 4950 rpm
Add lysis buffer	380 μL
Heat incubation	10 min at 56°C
Add ethanol	500 μL
Bind nucleic acids to TruTip matrix	20 pipetting cycles*
TruTip Wash 1	1000 μL; 10 pipetting cycles
TruTip Wash 2	1000 μL; 5 pipetting cycles
TruTip Wash 3	1000 μL; 5 pipetting cycles
Air dry TruTip matrix	45 sec forced air per TruTip
Elute purified DNA	500 μL, 10 pipetting cycles

* All pipetting operations were programmed at 130 μL sec^-1^ flow rate.

A Norgen Biotek Sputum DNA Purification Kit (Ontario, Canada; #46200) served as a reference extraction method to evaluate automated TruTip performance on simulated (spiked) sputum samples. Briefly, 1 mL of contrived sample was liquefied by adding an equal volume of 100 μg mL^-1^ dithiothreitol and heating at 37°C for 20 minutes. The entire volume of liquefied sample was then processed as per the manufacturer’s instructions, and purified DNA eluted in 100 μL elution buffer. Purified DNA was stored at -20°C until use.

### Quantitative IS6110 PCR

*M*. *tuberculosis*-specific DNA in nucleic acid extracts was amplified by real-time PCR (in duplicate) using a Roche Lightcycler 480 and the multi-copy IS6110 insertion element as a proxy for *M*. *tuberculosis* in the primary specimen [Note: *M*. *tuberculosis* clinical strains carry zero to 27 copies with 17 in the genome of the H37Ra strain used in development and LoD studies [31]] [32]. Five microliters of each nucleic acid extract was combined with 20 μL master mix in a 96-well plate to achieve a final reaction composition of [1X LightCycler® FastStart DNA Master HybProbe buffer and enzyme (Roche), 2.5 mM MgCl_2_, 0.45 μM forward primer (5'—GGG-TAG-CAG-ACC-TCA-CCT-ATG), 1.35 μM reverse primer (5'—AGC-GTA-GGC-GTC-GGT-GA), and 25 nM minor groove binding internal probe (5’ 6FAM-TCG-CCT-ACG-TGG-CCT-TT-MGB)]. Microtiter plates were loaded onto the thermal cycler, denatured for 10 min at 95°C, and cycled for 45 cycles of [95°C for 15 sec, 60°C for 60 sec]. A dilution series of H37Ra genomic DNA (from 10 pg μL^-1^–2.4 fg μL^-1^) was run at least monthly to verify amplification reagents and PCR efficacy, and an aliquot of one of the standard dilutions processed with each PCR plate as an external positive control. *M*. *tuberculosis* DNA was considered “detected” if the C_t_ value for both PCR replicates was ≤ 37.

## Results and discussion

The design principle for integrating a mechanical sample homogenizer with TruTip nucleic acid purification is described elsewhere [33]. Briefly, the concept involves separating the consumable from the energy-transducing mechanism–in this case, a rotating cylindrical magnet that is external to the lysis tubes (or reagent plate). At high rotational speeds, the external magnet induces the rotation of a small magnetic stir disc inside of each lysis tube, which in turn agitates the solid particles. Particle agitation is controlled by adjusting the speed of the external, rotating magnet. Magnetically-induced vortexing (MagVor) therefore generates a chaotic bead-milling or bead beating effect within the sample using simple hardware components and with minimal heat generated in the solution. Adjacent to the rotating magnet is a motorized carriage with integrated heater that holds all consumables for the extraction including sample lysis tubes, heater tubes, and a 96-well reagent plate. The carriage controls the position of the consumables relative to a fixed-position, 8-channel, custom-built pipetting manifold. The pipetting manifold (with attached TruTips) moves vertically up and down and a positive-displacement syringe pump controls the speed and aspiration/dispense volumes across all eight tips simultaneously. Pipetting speed is used as a proxy and means for controlling flow rate through the TruTip matrix. A selection valve and small air pump control air flow to the individual tips to dry the silica binding matrix after wash steps and before nucleic acid elution. Biosafety concerns were (partially) addressed in the prototype workstation by incorporating a HEPA-filtered exhaust fan and enclosure around the moving parts and consumables, creating a negative pressure air box relative to the operating environment. Electronic components were protected from potential liquid splashes by protective covers that can be wiped down with bleach or other disinfectants. The prototype was also designed to fit within a standard Biosafety cabinet if needed or desired.

The synergistic, positive impact of integrated bead milling on nucleic acid recovery is summarized in Table 2, where DNA yield at least doubled (> 1 ΔC_t_) at cell inputs ≥ 10^3^ CFU mL^-1^ (*p* < 0.05) and *M*. *tuberculosis* dilutions [at 100% extraction and detection efficiency, a 1 C_t_ difference represents a 100% increase in recovered DNA]. The data also show that the extra off-line enzymatic liquefaction step is not required for efficient DNA extraction, but was retained for all subsequent experiments because it simplified the transfer of a precise sputum volume from the primary sample container to the automated system lysis tubes. Given the heterogenous nature of individual sputum samples and an inability to accurately split a primary sputum sample, we were unable to determine the relative effect of MagVor alone on native (non-spiked) specimens.

**Table 2:** Relative impact of MagVor homogenization and lysis on *M*. *tuberculosis* DNA recovery from non-liquefied, raw sputum.

*M*. *tuberculosis* (CFU mL^-1^)	Average C_t_ ± St.Dev			
	No MagVor	With MagVor	ΔC_t_	*p*
10^4^	29.93 ± 0.48	28.47 ± 0.61	1.46	0.001
10^3^	31.20 ± 0.35	30.03 ± 0.55	1.17	0.03
10^2^	33.73 ± 1.10	32.64 ± 0.76	1.09	0.08
10^1^	35.26* ± 0.43	33.99* ± 1.27	1.27	0.33
0	ND	ND	N/A	N/A

Data represent the average from 4 replicate extractions and 2 real-time PCR tests per extract.

* Two PCR replicates from the n = 8 reactions were negative.

In the absence of a centrifugation step or a re-sealable lysis tube, there is a risk of generating aerosols during the homogenization step and contaminating other samples processed in parallel. To address the risk, 2 x 10^8^ cells of H37Ra were added to 12 independent sputum samples and processed in three replicate runs, where every other lysis tube, channel, and row in the reagent plate contained a water blank. There was no detectable IS6110 DNA in any of the water blanks (n = 36; C_t_ values > 37 at an estimated LoD of 3 fg per reaction). All other external negative controls processed during this work (n = 53 system runs containing negative controls) were likewise negative (not shown), indicating that the MagVor homogenization does not introduce a significant sample contamination risk.

**Fig 1:**
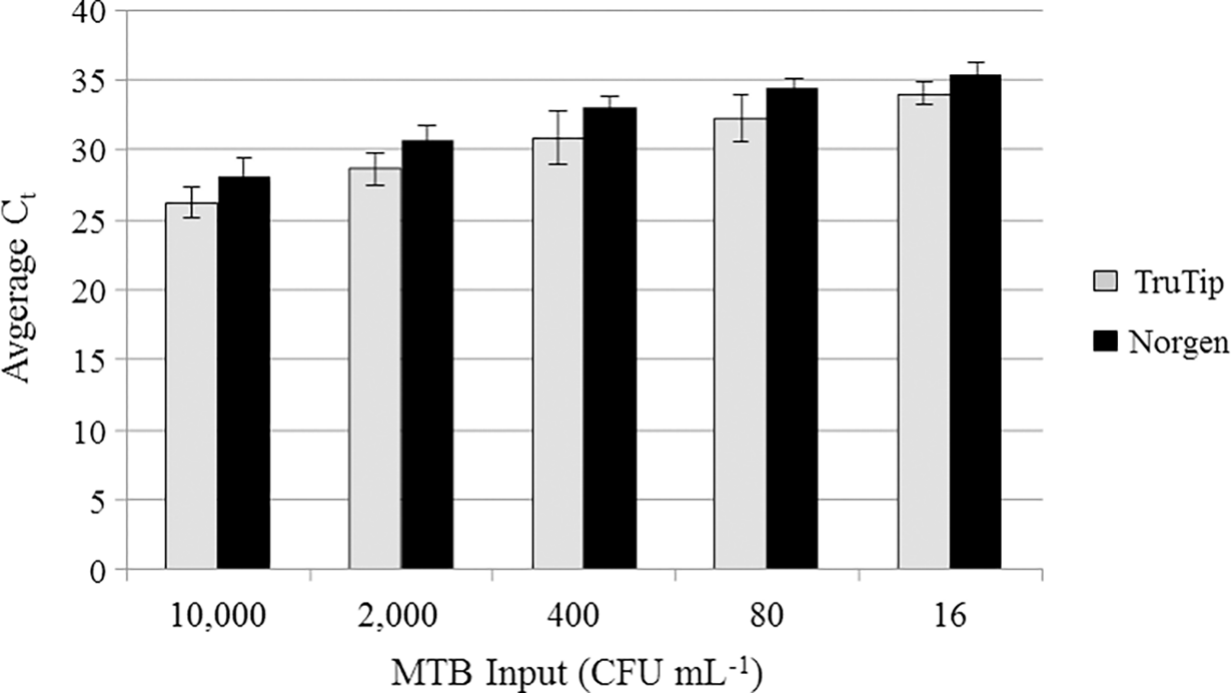
*M*. *tuberculosis* DNA recovery from spiked sputum. C_t_ values are the average of six independent sputa and two PCR tests per extract (n = 12 data points; *p* ≤ 0.05 for each dilution).

We established analytical performance metrics for the integrated TruTip workstation by amending six independent, TB-negative sputa with a five-fold dilution series of H37Ra cells and extracting them with the automated protocol in Table 1. Real-time PCR data (C_t_) values are summarized in Fig 1 (*p* ≤ 0.05 for each dilution). Because there was no fully automated sputum extraction method available at the time of this study, we used a commercially-available, manual Norgen Sputum DNA Isolation Kit as a benchmark of performance rather than a head-to-head comparator. One replicate extract from one of the six TruTip sputum samples was PCR-negative at 16 CFU mL^-1^ (PCR detection limit of 3 fg DNA (< 1 *M*. *tuberculosis* cell equivalent)). From these data, the automated workstation can repeatedly and reliably extract *M*. *tuberculosis*-detectable DNA from sputum (of variable background composition) containing between 16 and 80 CFU mL^-1^. The estimated limit of detection across multiple sputum backgrounds is consistent with published performance guidelines [2] and is within the analytical sensitivity range for detecting *M*. *tuberculosis* DNA in smear-negative specimens.

Clinical sample characteristics are summarized in S1 Table. Nine samples were both smear negative and culture negative, so these nucleic acid extraction and PCR amplification results are not included in the final analysis (below). Sputum samples ranged from saliva-like to hemoptoic (containing blood), with most samples characterized as mucoid (mucus-like) or mucopurulent (containing mucus and pus). One of the AFB smear positive (+), solid culture positive (+) sputum samples was consumed in its entirety during NALC-NaOH decontamination, leaving 123 sputum extracts and 124 sediment extracts for automated nucleic acid extraction.

The *M*. *tuberculosis* DNA detection rate was 99% and 100% for sputum and sediment, respectively, for all samples that were not categorized as smear negative, culture negative (Table 3). The single false-negative for sputum was at the detection limit for the paired sediment sample and was positive upon re-extraction. There was no significant relationship between sample quality and *M*. *tuberculosis* DNA detection efficacy, indicating that the automated TruTip protocol and workstation is equally effective regardless of sputum composition or consistency. Although only a few samples in the clinical set were described as containing blood, the procedure and reagents are similar to that used for extraction from whole blood, which results in high purity nucleic acid without PCR inhibitors. Even so, additional studies on a wider range of sputum types are required to further characterize the protocol performance.

**Table 3:** *M*. *tuberculosis* DNA detection efficacy relative to primary sample characteristics.

	*M*. *tuberculosis* DNA Detection Rate	
Sample Characteristic	Sputum	Sediment
AFB smear positive, culture positive	109/109	110/110
AFB smear negative, culture positive	8*/9	9/9
AFB smear positive, culture negative	5/5	5/5
Total	122/123	124/124
	99% (95% CI 96–100)	100% (95% CI 97–100)

Extracts generating real time C_t_ values ≥ 37 are considered Indeterminate for *M*. *tuberculosis* DNA and were confirmed negative by re-running the PCR test. Primary data are found in S1 Table.

*The one missed sample was positive when re-extracted.

DNA recovery relative to AFB smear and culture status is summarized in Table 4. There was a significant linear correlation between DNA recovery and smear status (or quantity of acid fast bacteria) for both sputum and sediment extracts, which corresponds with the estimated bacillary load in the original sample. Nucleic acid recovery was significantly higher from raw sputum as compared to that of matched sediments regardless of smear or culture grade (inclusive of smear negative and culture negative samples), averaging at least five times (log_2_ 2.45) more DNA recovered from raw sputum than sediment (*p* < 0.0001) and > 10X yield in 34% (39/116) of the smear positive specimens. In fact, there were only 14 samples where there was more detectable *M*. *tuberculosis* DNA in the sediment extract than in the raw sputum extract (S1 Table, negative ΔC_t_ values). We suspect that the lower DNA recovery from sediment is due, in part, to cell and/or DNA loss during the sputum decontamination and sedimentation process (e.g., [34]), or minimal amounts of human DNA in sediment that may otherwise serve as a carrier in sputum extracts. Nevertheless, the data show that one and the same automated protocol is equally efficacious on raw sputum and sediment, regardless of sputum quality or characteristics, and that primary sputum may actually be the *preferred* specimen for sensitive *M*. *tuberculosis* detection or diagnosis.

**Table 4:** Estimated *M*. *tuberculosis* DNA recovery from raw sputum or sediment relative to AFB smear or solid culture grade.

		Average C_t_		Average ΔC_t_^b^	*p* value
AFB Smear Grade	N	Sputum	Sediment		
+ 1	31	25.80 ± 3.50	28.99 ± 3.83	3.49	< 0.0001
+ 2	34	22.41 ± 2.04	25.23 ± 2.12	2.82	< 0.0001
+ 3	53 sputum / 54 sediments	20.36 ± 2.36	22.32 ± 1.99	1.95	< 0.0001
*R*^*2*^		0.9803	0.9947		
Solid Culture Grade^a^					
+ 1	40 sputum / 41 sediments	23.72 ± 4.08	26.60 ± 4.71	2.98	<0.0001
+ 2	46	22.17 ± 2.55	24.58 ± 3.32	2.41	< 0.0001
+ 3	27	20.58 ± 2.69	23.05 ± 2.84	2.47	< 0.0001
*R*^*2*^		0.9999	0.9938		

Any detectable acid fast bacteria or colony (scanty designation) was counted as a +1 AFB smear or +1 culture sample, respectively.

^a^ Cultures were graded as: + 1 = 1 to 100 colonies; +2 = 100 to 200 colonies; and + 3 > 200 colonies.

^b^ ΔC_t_ is calculated as Ave C_t_ (sediment)–Ave C_t_ (sputum). Assuming 100% extraction and PCR efficiency, ΔC_t_ = 3.32 corresponds to a 10-fold difference in DNA recovery.

**Fig 2:**
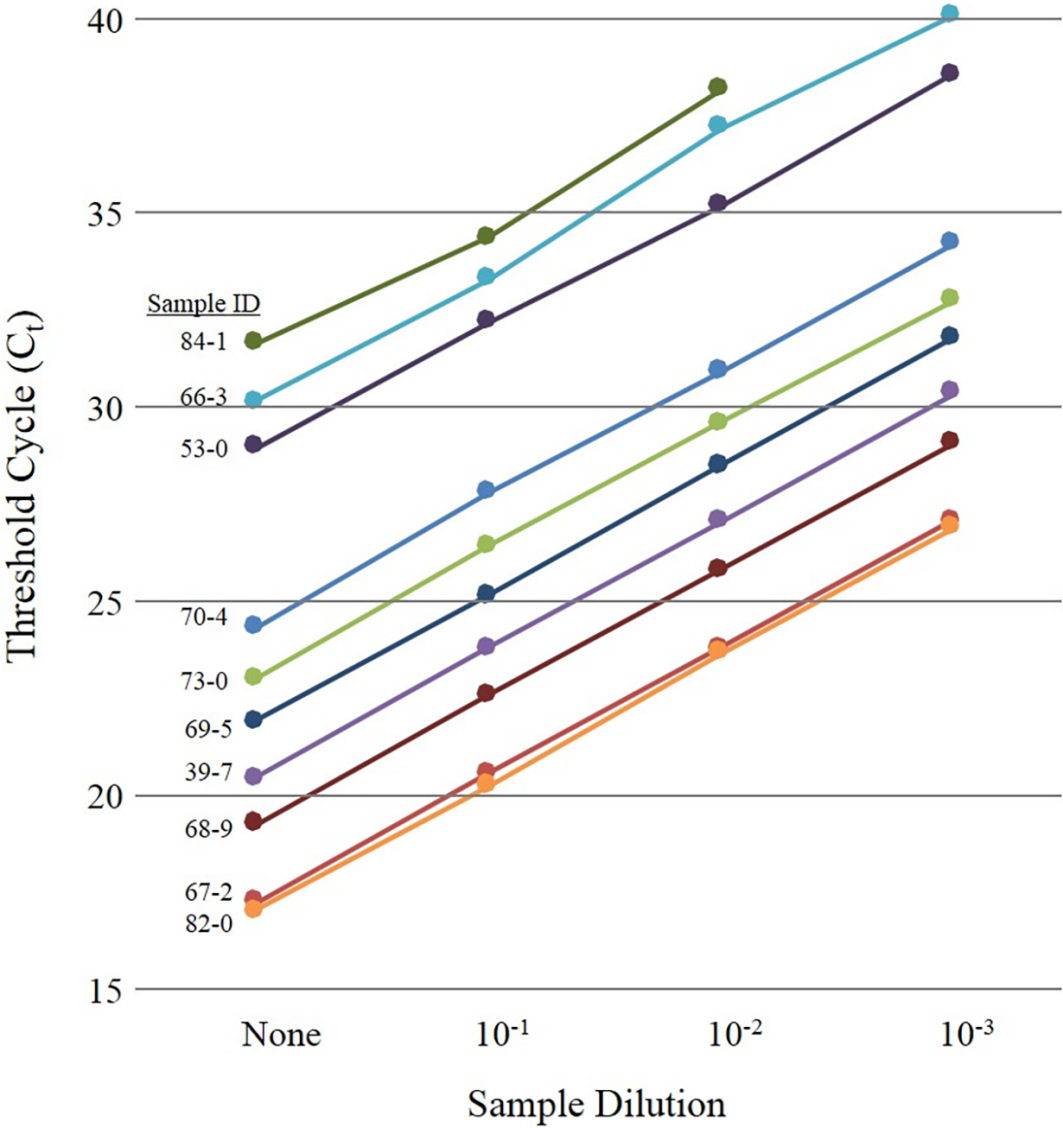
Dilution of sputum extracts to investigate inhibition. Average C_t_ value of serially diluted sputum extracts for selected samples with variable quality characteristics (n = 3 for each C_t_ value; R^2^ > 0.997 for each sample).

While Table 4 shows a strong correlation between AFB smear status and average C_t_ value (or DNA recovery), we nevertheless tested for the possibility that the sputum extracts contained PCR (or fluorescence) inhibitors that affect the accuracy of the C_t_ measurements. Ten independent sputum extracts of variable quality characteristics were diluted in ultra-pure water and re-analyzed by real-time PCR, with data summarized in Fig 2. The correlation coefficient (R^2^) was > 0.997 for each sample (not shown). A separate experiment with purified *M*. *tuberculosis* DNA amended into PCR-grade water or a pooled, TB-negative sputum extract also showed no difference in C_t_ values over a range of DNA input concentrations (not shown). In combination, these data indicate that amplification or fluorescent inhibitors (if present) were not detectable or negatively influencing reported C_t_ values over the course of this study.

We should point out that our study used retrospective frozen specimens which could prematurely break down sputum and lyse bacterial cell walls. Because we focused on challenging the system with the most viscous sputum from smear positive TB patients, we acknowledge that further study including TruTip efficacy on prospective (fresh) specimens with statistically relevant prevalence of smear-negative specimens is required to accurately estimate the clinical sensitivity.

The long-term technology objective of this research is to unite automated sputum sample preparation with a *M*. *tuberculosis* drug susceptibility test (e.g., [35]) in a flexible, sample-to-answer diagnostic device. We opted to pursue an “open” sample preparation fluidic architecture of TruTips and reagent plates because the approach (and workstation) provides several straightforward opportunities to increase input sample size (volume), modify reagents, and/or adjust procedural steps to increase extraction efficacy, if needed. Indeed, the specific design of the automated workstation and its open fluidic architecture provides a level of engineering and IVD product development flexibility that is difficult to achieve with cartridge-based or otherwise “closed”, point-of-care microfluidic devices. As a stand-alone workstation, however, there is now an opportunity to push sputum nucleic acid sample preparation closer to the point of need and independent of any specific nucleic acid detection technology.

TruTip protocols can also be automated on a personal, electronic pipette (e.g., [28]), so we might expect a similar level of performance by translating the sputum protocol to an on-demand, more manual format and a set of sample preparation tools. Conversely, prior nasopharyngeal aspirate, swab, blood, plasma and saliva protocols [28, 30, 36–38] can be easily transferred to the automated workstation reported here with integrated heater to further simplify chemical lysis and enzymatic procedures typically performed off-line. TruTip therefore satisfies a basic sample processing demand identified by Denkinger [4] and others [2, 20, 39, 40]. At the same time, the ability to transfer protocols between personalized, on-demand TruTip tools and fully automated workstation affords an opportunity to standardize nucleic acid sample preparation procedures, reagents, and quality control across multiple levels of the health care system, irrespective of desired throughput. We therefore expect TruTip and the automated workstation will add value to the TB community as a stand-alone sample preparation device while continued research focuses on integrating automated TruTip sample preparation with drug-resistant TB amplification microarrays.

## Supporting information

S1 Table Clinical characteristics and raw real-time data for sputum specimens. (DOCX)
