# Supplementary material for: Automated TruTip nucleic acid extraction and purification from raw sputum
Source: PLoS One. 2018 Jul 5;13(7):e0199869. doi: 10.1371/journal.pone.0199869 (PMC6033430; doi:10.1371/journal.pone.0199869)
Supplement: S1 Table — (DOCX) [file pone.0199869.s001.docx]

S1 Table. Clinical characteristics and raw real-time data for sputum specimens.

| Sample ID | Sample Quality | Visible Appearance before TruTip Extraction | AFB Smear Status^1^ | Solid Culture Grade^2^ | Automated TruTip Extraction - IS6110 Real-Time PCR Results (C_t_) | | | | | | |
| --- | --- | --- | --- | --- | --- | --- | --- | --- | --- | --- | --- |
|  |  |  |  |  | Raw Sputum | |  | Decontaminated Sediment | | | ∆C_t_^3^ |
|  |  |  |  |  | Replicate 1 | Replicate 2 | Average C_t_ | Replicate 1 | Replicate 2 | Average C_t_ |  |
| 63 | Mucoid | Clear and yellow | + 1 | + 1 | 27.81 | 27.87 | 27.84 | 31.37 | 31.29 | 31.33 | 3.49 |
| 76 | Salival | Light yellow | + 1 | + 1 | 27.13 | 27.04 | 27.09 | 31.73 | 31.68 | 31.71 | 4.62 |
| 82 | Salival | Pale yellow and cloudy | + 1 | + 1 | 24.81 | 24.85 | 24.83 | 25.96 | 25.91 | 25.94 | 1.11 |
| 120 | Salival | Clear | + 1 | + 1 | 26.69 | 26.70 | 26.70 | 32.12 | 32.18 | 32.15 | 5.46 |
| 123 | Mucoid | Clear | + 1 | + 1 | 27.87 | 27.91 | 27.89 | 30.41 | 30.48 | 30.45 | 2.56 |
| 66 | Salival | Clear | + 1 | + 1 | 30.14 | 30.07 | 30.11 | 34.68 | 34.86 | 34.77 | 4.67 |
| 97 | Salival | Clear | + 1 | + 1 | 28.75 | 28.78 | 28.77 | 27.73 | 27.78 | 27.76 | -1.01 |
| 98 | Mucopurulent | Thick and yellow | + 1 | + 1 | 22.69 | 22.74 | 22.72 | 26.55 | 26.62 | 26.59 | 3.87 |
| 111 | Mucoid | Brown | + 1 | + 1 | 24.77 | 25.16 | 24.97 | 27.34 | 27.41 | 27.38 | 2.41 |
| 6 | Mucopurulent | ND | + 1 | + 2 | 19.02 | 18.94 | 18.98 | 28.50 | 28.55 | 28.53 | 9.55 |
| 34 | Salival | White and cloudy | + 1 | + 2 | 22.49 | 22.59 | 22.54 | 25.41 | 25.42 | 25.42 | 2.88 |
| 44 | Mucoid | Light yellow | + 1 | + 2 | 24.66 | 24.67 | 24.67 | 27.07 | 27.13 | 27.10 | 2.44 |
| 52 | Mucoid | Clear with a few chunks | + 1 | + 2 | 22.22 | 22.16 | 22.19 | 24.18 | 24.23 | 24.21 | 2.02 |
| 70 | Mucopurulent | Cloudy | + 1 | + 2 | 24.64 | 24.51 | 24.58 | 27.93 | 27.95 | 27.94 | 3.36 |
| 71 | Salival | Clear | + 1 | + 2 | 25.46 | 25.42 | 25.44 | 21.81 | 21.83 | 21.82 | -3.62 |
| 72 | Mucoid | Clear | + 1 | + 2 | 22.92 | 22.97 | 22.95 | 30.59 | 30.58 | 30.59 | 7.64 |
| 73 | Mucoid | Pale yellow and cloudy | + 1 | + 2 | 23.16 | 23.00 | 23.08 | 26.30 | 26.27 | 26.29 | 3.21 |
| 89 | Mucoid | Yellow and cloudy | + 1 | + 2 | 20.67 | 20.59 | 20.63 | 22.84 | 22.79 | 22.82 | 2.19 |
| 112 | Mucoid | Clear | + 1 | + 2 | 24.71 | 24.66 | 24.69 | 36.24 | 35.80 | 36.02 | 11.34 |
| 129 | Mucoid | Thick, yellow, and cloudy | + 1 | + 2 | 22.24 | 23.50 | 22.87 | 25.01 | 24.97 | 24.99 | 2.12 |
| 35 | Mucopurulent | Pale yellow | + 1 | + 2 | 27.22 | 27.47 | 27.35 | 33.35 | 33.59 | 33.47 | 6.13 |
| 14 | Mucoid | Yellow with small mucus chunk | + 1 | + 3 | 22.55 | 22.56 | 22.56 | 27.05 | 26.97 | 27.01 | 4.46 |
| 107 | Salival | Clear | + 1 | + 3 | 27.47 | 27.40 | 27.44 | 30.68 | 30.66 | 30.67 | 3.24 |
| 124 | Salival | Clear | + 1 | + 3 | 25.08 | 25.07 | 25.08 | 25.28 | 25.18 | 25.23 | 0.16 |
| 20 | Mucoid | Pale yellow with small mucus chunk | + 2 | + 1 | 23.64 | 23.69 | 23.67 | 23.78 | 23.83 | 23.81 | 0.14 |
| 23 | Mucopurulent | Pale yellow and cloudy | + 2 | + 1 | 20.37 | 20.40 | 20.39 | 23.74 | 23.74 | 23.74 | 3.36 |
| 27 | Mucopurulent | Clear | + 2 | + 1 | 21.93 | 21.92 | 21.93 | 25.83 | 25.82 | 25.83 | 3.90 |
| 32 | Mucoid | Pale yellow with small mucus chunk | + 2 | + 1 | 23.58 | 23.43 | 23.51 | 27.25 | 27.27 | 27.26 | 3.76 |
| 36 | Hemoptoic | Bloody and cloudy | + 2 | + 1 | 20.63 | 20.69 | 20.66 | 31.00 | 30.87 | 30.94 | 10.28 |
| 47 | Mucoid | Yellow with lvery large mucus chunk | + 2 | + 1 | 25.49 | 25.52 | 25.51 | 26.84 | 26.92 | 26.88 | 1.38 |
| 56 | Mucopurulent | Clear with large mucus chunk | + 2 | + 1 | 19.70 | 19.74 | 19.72 | 23.54 | 23.40 | 23.47 | 3.75 |
| 94 | Purulent | Yellow and cloudy | + 2 | + 1 | 22.02 | 22.09 | 22.06 | 22.62 | 22.63 | 22.63 | 0.57 |
| 121 | Mucoid | Light yellow | + 2 | + 1 | 21.04 | 21.05 | 21.05 | 26.34 | 26.32 | 26.33 | 5.29 |
| 13 | Mucoid | Thin and clear | + 2 | + 2 | 24.55 | 24.51 | 24.53 | 26.95 | 26.98 | 26.97 | 2.44 |
| 22 | Salival | Thin and clear | + 2 | + 2 | 21.49 | 21.32 | 21.41 | 23.35 | 23.16 | 23.26 | 1.85 |
| 39 | Mucoid | Light yellow and cloudy | + 2 | + 2 | 20.34 | 20.35 | 20.35 | 22.06 | 22.07 | 22.07 | 1.72 |
| 41 | Mucoid | White and cloudy | + 2 | + 2 | 22.15 | 22.17 | 22.16 | 23.75 | 23.81 | 23.78 | 1.62 |
| 43 | Mucoid | Thick, yellow, and cloudy | + 2 | + 2 | 21.27 | 21.32 | 21.30 | 25.03 | 25.00 | 25.02 | 3.72 |
| 46 | Mucopurulent | Light yellow | + 2 | + 2 | 25.67 | 25.65 | 25.66 | 22.60 | 22.53 | 22.57 | -3.10 |
| 64 | Mucoid | Pale yellow | + 2 | + 2 | 24.76 | 24.79 | 24.78 | 25.60 | 25.62 | 25.61 | 0.84 |
| 69 | Mucoid | Clear and light yellow | + 2 | + 2 | 21.75 | 21.76 | 21.76 | 24.17 | 24.21 | 24.19 | 2.44 |
| 77 | Salival | Cloudy | + 2 | + 2 | 24.41 | 24.41 | 24.41 | 27.26 | 27.35 | 27.31 | 2.90 |
| 78 | Mucopurulent | Thick, dark yellow, and cloudy | + 2 | + 2 | 21.13 | 20.95 | 21.04 | 25.87 | 26.16 | 26.02 | 4.98 |
| 84 | Salival | Clear and light yellow | + 2 | + 2 | 24.16 | 24.16 | 24.16 | 26.41 | 26.41 | 26.41 | 2.25 |
| 85 | Mucoid | ND | + 2 | + 2 | 27.56 | 27.62 | 27.59 | 30.31 | 30.37 | 30.34 | 2.75 |
| 87 | Salival | Thick and yellow | + 2 | + 2 | 21.66 | 21.68 | 21.67 | 24.95 | 24.98 | 24.97 | 3.30 |
| 99 | Mucopurulent | Light yellow | + 2 | + 2 | 22.64 | 22.57 | 22.61 | 25.37 | 25.27 | 25.32 | 2.72 |
| 106 | Mucopurulent | Thick and dark yellow | + 2 | + 2 | 19.01 | 19.04 | 19.03 | 25.02 | 25.02 | 25.02 | 6.00 |
| 115 | Mucoid | cloudy | + 2 | + 2 | 21.72 | 21.77 | 21.75 | 23.82 | 23.84 | 23.83 | 2.09 |
| 18 | Mucoid | Thin and clear | + 2 | + 3 | 22.99 | 23.00 | 23.00 | 25.34 | 25.32 | 25.33 | 2.34 |
| 59 | Mucoid | Light yellow with mucus chunk | + 2 | + 3 | 23.41 | 23.48 | 23.45 | 26.24 | 26.20 | 26.22 | 2.78 |
| 101 | Mucopurulent | Yellow and cloudy | + 2 | + 3 | 19.17 | 19.09 | 19.13 | 20.85 | 20.81 | 20.83 | 1.70 |
| 108 | Mucopurulent | Thick and dark yellow | + 2 | + 3 | 18.82 | 18.81 | 18.82 | 23.00 | 22.99 | 23.00 | 4.18 |
| 122 | Salival | Yellow | + 2 | + 3 | 23.81 | 23.78 | 23.80 | 25.77 | 25.80 | 25.79 | 1.99 |
| 17 | Mucoid | Yellow and cloudy | + 3 | + 1 | 15.83 | 15.93 | 15.88 | 19.80 | 19.77 | 19.79 | 3.91 |
| 19 | Mucoid | Yellow with small mucus chunks | + 3 | + 1 | 22.55 | 22.59 | 22.57 | 22.14 | 22.21 | 22.18 | -0.40 |
| 24 | Salival | Clear | + 3 | + 1 | 21.39 | 21.35 | 21.37 | 23.57 | 23.57 | 23.57 | 2.20 |
| 28 | Mucoid | Pale yellow and cloudy | + 3 | + 1 | 19.92 | 19.99 | 19.96 | 22.75 | 22.71 | 22.73 | 2.78 |
| 29 | Salival | White and cloudy | + 3 | + 1 | 18.66 | 18.63 | 18.65 | 20.78 | 20.74 | 20.76 | 2.12 |
| 51 | Salival | Light yellow with a large mucus cluster | + 3 | + 1 | 20.43 | 19.51 | 19.97 | 25.59 | 25.53 | 25.56 | 5.59 |
| 58 | Salival | Very thick and yellow | + 3 | + 1 | 21.24 | 21.23 | 21.24 | 20.01 | 19.93 | 19.97 | -1.27 |
| 74 | Mucoid | Yellow | + 3 | + 1 | 21.91 | 21.87 | 21.89 | 25.85 | 25.93 | 25.89 | 4.00 |
| 80 | Salival | Pale yellow and cloudy | + 3 | + 1 | 21.96 | 21.11 | 21.54 | 23.18 | 23.12 | 23.15 | 1.62 |
| 95 | Mucoid | Clear and light yellow | + 3 | + 1 | 26.57 | 26.52 | 26.55 | 24.93 | 25.02 | 24.98 | -1.57 |
| 116 | Mucoid | Pale yellow | + 3 | + 1 | 20.51 | 20.56 | 20.54 | 23.19 | 23.26 | 23.23 | 2.69 |
| 118 | Mucoid | Light yellow and cloudy | + 3 | + 1 | 21.63 | 21.55 | 21.59 | 24.91 | 24.92 | 24.92 | 3.33 |
| 127 | Salival | Light yellow | + 3 | + 1 | 20.09 | 20.22 | 20.16 | 22.17 | 22.14 | 22.16 | 2.00 |
| 60 | Mucopurulent | Yellow with two large chunks | + 3 | + 1 | 18.03 | 18.06 | 18.05 | 18.31 | 18.32 | 18.32 | 0.27 |
| 92 | Mucoid | Thick and dark yellow | + 3 | + 1 | 18.27 | 18.15 | 18.21 | 21.73 | 21.76 | 21.75 | 3.54 |
| 105 | Purulent | ND | + 3 | + 1 | N/A | N/A | N/A | 22.78 | 22.69 | 22.74 | N/A |
| 1 | Mucopurulent | Clear mucus | + 3 | + 2 | 25.66 | 25.71 | 25.69 | 23.01 | 23.09 | 23.05 | -2.64 |
| 5 | Mucopurulent | ND | + 3 | + 2 | 24.63 | 24.58 | 24.61 | 21.56 | 21.56 | 21.56 | -3.05 |
| 9 | Mucopurulent | ND | + 3 | + 2 | 20.95 | 20.93 | 20.94 | 21.90 | 22.08 | 21.99 | 1.05 |
| 16 | Mucopurulent | Dark yellow with small mucus chunk | + 3 | + 2 | 18.14 | 18.19 | 18.17 | 20.96 | 20.96 | 20.96 | 2.80 |
| 25 | Purulent | Very thick, dark yellow-to-green | + 3 | + 2 | 23.87 | 23.71 | 23.79 | 21.13 | 21.06 | 21.10 | -2.70 |
| 37 | Mucoid | Thin and yellow | + 3 | + 2 | 20.68 | 20.72 | 20.70 | 23.39 | 23.39 | 23.39 | 2.69 |
| 40 | Salival | Thin and pale-to-dark yellow | + 3 | + 2 | 19.43 | 19.49 | 19.46 | 20.47 | 20.54 | 20.51 | 1.05 |
| 42 | Mucoid | White and cloudy | + 3 | + 2 | 21.91 | 21.92 | 21.92 | 23.59 | 23.63 | 23.61 | 1.70 |
| 48 | Mucopurulent | Yellow/green and mucoid | + 3 | + 2 | 25.90 | 25.88 | 25.89 | 21.55 | 21.46 | 21.51 | -4.39 |
| 50 | Mucoid | Light yellow with mucus chunks | + 3 | + 2 | 20.04 | 20.04 | 20.04 | 23.32 | 23.34 | 23.33 | 3.29 |
| 54 | Mucopurulent | Yellow, very mucoid | + 3 | + 2 | 19.70 | 19.74 | 19.72 | 22.08 | 22.11 | 22.10 | 2.38 |
| 55 | Salival | Pale yellow | + 3 | + 2 | 21.24 | 21.27 | 21.26 | 24.03 | 24.04 | 24.04 | 2.78 |
| 67 | Mucopurulent | Thick, yellow, and cloudy | + 3 | + 2 | 17.41 | 17.18 | 17.30 | 19.55 | 19.57 | 19.56 | 2.27 |
| 68 | Purulent | Pale yellow | + 3 | + 2 | 19.07 | 19.02 | 19.05 | 23.77 | 23.77 | 23.77 | 4.73 |
| 109 | Purulent | Thick and yellow | + 3 | + 2 | 18.33 | 18.37 | 18.35 | 21.94 | 21.84 | 21.89 | 3.54 |
| 110 | Mucoid | Thick and dark yellow | + 3 | + 2 | 20.39 | 20.51 | 20.45 | 24.49 | 24.62 | 24.56 | 4.11 |
| 113 | Mucoid | Light yellow | + 3 | + 2 | 20.39 | 20.37 | 20.38 | 21.77 | 21.68 | 21.73 | 1.35 |
| 133 | Mucopurulent | Yellow | + 3 | + 2 | 18.02 | 17.94 | 17.98 | 20.18 | 20.24 | 20.21 | 2.23 |
| 2 | Mucopurulent | ND | + 3 | + 3 | 17.13 | 17.16 | 17.15 | 20.82 | 20.81 | 20.82 | 3.67 |
| 3 | Mucopurulent | ND | + 3 | + 3 | 18.40 | 18.32 | 18.36 | 22.09 | 22.13 | 22.11 | 3.75 |
| 4 | Mucopurulent | Very thick, yellow, and mucoid | + 3 | + 3 | 20.00 | 20.01 | 20.01 | 20.79 | 20.77 | 20.78 | 0.77 |
| 10 | Mucoid | Thin and clear | + 3 | + 3 | 20.68 | 20.58 | 20.63 | 23.65 | 23.78 | 23.72 | 3.09 |
| 12 | Mucoid | Thin and yellow | + 3 | + 3 | 20.16 | 20.14 | 20.15 | 22.26 | 22.23 | 22.25 | 2.10 |
| 15 | Mucoid | ND | + 3 | + 3 | 18.23 | 18.15 | 18.19 | 19.13 | 19.22 | 19.18 | 0.98 |
| 31 | Purulent | Dark yellow and very, very thick with large chunks | + 3 | + 3 | 19.53 | 19.51 | 19.52 | 23.08 | 23.11 | 23.10 | 3.58 |
| 61 | Mucoid | Thick, yellow, with a large chunk | + 3 | + 3 | 15.14 | 15.10 | 15.12 | 16.68 | 16.65 | 16.67 | 1.55 |
| 62 | Mucoid | Thick, yellow, and cloudy | + 3 | + 3 | 20.03 | 20.04 | 20.04 | 23.38 | 23.33 | 23.36 | 3.32 |
| 65 | Mucoid | Thick and yellow | + 3 | + 3 | 19.18 | 19.14 | 19.16 | 22.00 | 22.04 | 22.02 | 2.86 |
| 79 | Purulent | Clear and yellow | + 3 | + 3 | 20.71 | 20.77 | 20.74 | 22.80 | 22.88 | 22.84 | 2.10 |
| 81 | Mucopurulent | Thick and dark yellow | + 3 | + 3 | 16.81 | 16.79 | 16.80 | 18.48 | 18.50 | 18.49 | 1.69 |
| 88 | Mucopurulent | Thick and dark yellow | + 3 | + 3 | 23.41 | 23.40 | 23.41 | 21.38 | 21.51 | 21.45 | -1.96 |
| 90 | Mucoid | Yellow and cloudy | + 3 | + 3 | 22.07 | 22.09 | 22.08 | 24.55 | 24.62 | 24.59 | 2.51 |
| 91 | Purulent | Thick and dark yellow | + 3 | + 3 | 19.79 | 19.79 | 19.79 | 24.38 | 24.42 | 24.40 | 4.61 |
| 96 | Mucopurulent | Thick and dark yellow | + 3 | + 3 | 19.14 | 18.99 | 19.07 | 21.42 | 21.17 | 21.30 | 2.23 |
| 125 | Mucopurulent | Thick, yellow, and cloudy | + 3 | + 3 | 19.15 | 19.10 | 19.13 | 22.59 | 22.58 | 22.59 | 3.46 |
| 128 | Salival | Clear and light yellow | + 3 | + 3 | 22.73 | 22.81 | 22.77 | 25.83 | 25.81 | 25.82 | 3.05 |
| 132 | Mucopurulent | Yellow and cloudy | + 3 | + 3 | 20.30 | 20.33 | 20.32 | 22.78 | 22.72 | 22.75 | 2.44 |
| 49 | Mucoid | White and cloudy | + 1 | Negative | 26.81 | 26.79 | 26.80 | 29.20 | 29.20 | 29.20 | 2.40 |
| 83 | Salival | Clear and light yellow | + 1 | Negative | 31.54 | 31.50 | 31.52 | 32.54 | 32.67 | 32.61 | 1.09 |
| 102 | Mucopurulent | Light yellow | + 1 | Negative | 32.90 | 32.79 | 32.85 | 37.17 | 37.26 | 37.22 | 4.37 |
| 114 | Mucoid | Light yellow | + 1 | Negative | 34.89 | 34.71 | 34.80 | 29.00 | 28.88 | 28.94 | -5.86 |
| 33 | Mucopurulent | Yellow with large mucus chunk | + 2 | Negative | 23.88 | 23.91 | 23.90 | 23.91 | 23.92 | 23.92 | 0.02 |
| 75 | Mucoid | Clear and light yellow | + 2 | Negative | 22.19 | 22.20 | 22.20 | 26.39 | 26.55 | 26.47 | 4.28 |
| 86 | Mucopurulent | Yellow and cloudy | + 2 | Negative | 24.17 | 24.15 | 24.16 | 27.28 | 27.33 | 27.31 | 3.15 |
| 117 | Salival | Light yellow and cloudy | + 2 | Negative | 21.06 | 21.00 | 21.03 | 25.51 | 25.58 | 25.55 | 4.52 |
| 100 | Mucoid | Thick and dark yellow | + 3 | Negative | 22.87 | 22.85 | 22.86 | 26.42 | 26.41 | 26.42 | 3.56 |
| 131 | Mucoid | Cloudy | Negative | + 1 | 28.19 | 28.15 | 28.17 | 33.72 | 33.74 | 33.73 | 5.56 |
| 8 | Salival | ND | Negative | + 1 | 32.29 | 32.27 | 32.28 | 34.35 | 34.47 | 34.41 | 2.13 |
| 26 | Mucoid | Clear with small mucus chunk | Negative | + 1 | 30.62 | 30.67 | 30.65 | 35.91 | 35.31 | 35.61 | 4.97 |
| 45 | Mucoid | Thin and cloudy | Negative | + 1 | 32.06 | 31.98 | 32.02 | 35.26 | 35.49 | 35.38 | 3.36 |
| 93 | Salival | Clear and light yellow | Negative | + 1 | ND | ND | ND | 36.68 | ND | 36.68 | N/A |
| 11 | Mucoid | Thin and light yellow | Negative | Negative | 33.37 | 33.24 | 33.31 | 36.65 | ND | 36.65 | 3.35 |
| 30 | Mucoid | Light yellow with small mucus chunks | Negative | Negative | 31.92 | 31.91 | 31.92 | 34.74 | 34.74 | 34.74 | 2.83 |
| 38 | Mucoid | Light yellow | Negative | Negative | 27.28 | 27.41 | 27.35 | 31.73 | 31.69 | 31.71 | 4.37 |
| 57 | Mucoid | Light yellow | Negative | Negative | 24.81 | 24.89 | 24.85 | 25.49 | 25.47 | 25.48 | 0.63 |
| 103 | Salival | Clear | Negative | Negative | 31.85 | 31.75 | 31.80 | 27.16 | 27.17 | 27.17 | -4.64 |
| 104 | Mucoid | Pale yellow and cloudy | Negative | Negative | 34.33 | 34.13 | 34.23 | 32.23 | ND | 32.23 | -2.00 |
| 119 | Salival | Clear | Negative | Negative | ND | ND | ND | 36.33 | 36.96 | 36.65 | N/A |
| 126 | Mucoid | Pale yellow | Negative | Negative | 29.54 | 29.47 | 29.51 | 32.22 | 31.95 | 32.09 | 2.58 |
| 130 | Mucoid | Thin and cloudy | Negative | Negative | 35.49 | 35.19 | 35.34 | ND | ND | ND | N/A |

N/A = not applicable

ND = not determined, or not detected

^1^ Any detectable acid fast bacteria was designated an AFB + 1 sample.

^2^ Solid cultures were graded as: Negative = no detectable colonies; + 1 = 1 to 100 colonies; +2 = 100 to 200 colonies; and + 3 > 200 colonies.

^3^ ∆C_t_ is calculated as Ave C_t_ (sediment) – Ave C_t_ (sputum). Assuming 100% extraction and PCR efficiency, ∆C_t_ = 3.32 corresponds to a 10-fold difference in DNA recovery.
